# On the Cellular Vitality of the Tissues, as an Evidence of Life in New-Born Infants

**Published:** 1866-09

**Authors:** B. H. Cheney

**Affiliations:** Joliet, Ill.


### ON THE CELLULAR VITALITY OF THE TISSUES, AS AN EVIDENCE OF LIFE IN NEW-BORN INFANTS.

By B. H. CHENEY, M.D., of Joliet, Ill.

It sometimes occurs that contraction of various muscles is observed in cases of new-born infants, who would otherwise be considered as still-born, there being no other sign of life. In such cases, physiological evidence seems conclusively to prove that such contractions are but the manifestation, on the application of due stimulus, of a property inherent, not only in the muscular fibre, but in all organized tissues. DALTON says: "Every organ in the body is endowed with the property of *irritability;* that is, the property of reacting in some peculiar manner, when subjected to the action of a direct stimulus."* Such stimuli may well be, in the case of a tender, new-born infant, the external air, water, or any medium or substance hitherto foreign to its life, but to which it is subjected on coming into the world.

In reference to this property of irritability as regards muscle, it is expressly stated by the same author, that if the heart of a warm-blooded animal be rapidly removed from the chest, and, *when its pulsations have ceased,* touched with the point of a steel needle, its contractions can be readily excited.† It will not be contended that any other life than that peculiar to organized structures, and inherent therein, is manifested thereby.‡ We find the same property, namely, that of acting in accordance with a particular function, respondent to due and direct stimulus, to exist also in the nerve-fibre, so long as its continuity remains unbroken, and there is still enough of its molecular (cellular) vitality left to render it organized. By molecular (cellular) vitality, is to be understood simply that life which exists in every organized structure, so long as nutrition is going on therein.§

It is necessary, however, to observe, in this connection, the distinction between *organized* and *organic.* "Each substance called into existence, (manifested,) in the life of an organism,

* Human Physiology, p. 370.

† Dalton, *opus citat.* p. 57, *et seq.*

‡ "By the irritability of tissues is simply meant a capability of receiving impressions from surrounding agents, and thus producing phenomena, and is only to be observed when these tissues are alive. It is the 'capability of being acted upon.' It, of course, belongs to everything that has life; to plants as well as animals; to the organic molecular cell, as truly as to the most perfect and complicated structure." [Hodge, Dis. of Women, p. 19.

§ "Every animal presents itself as a sum of vital unities." [Virchow, Cell. Path. p. 40.

which is not found in the inorganic world, is called organic; but it does not follow that it must necessarily be organized. That is, it may appear to the eye homologous, and be incapable of separation into dissimilar parts by the knife, or by other anatomical means.

"Everything organized, on the contrary, consists of several organic substances of certain form, each one of which possesses certain peculiarities, which influence each other according to certain laws, and show themselves by dissection, or by the microscope, to be different.

"Albumen, protein, serum of blood, etc., are organic, but not organized: they are called, therefore, organic substances without form. Nerve, muscle, gland-tissue, etc., are, on the other hand, organized, and, *eo ipso*, organic."*

Physiological life, then, or the molecular (cellular) vitality resident in every organized tissue in a state of nutrition, is common to all, and is, in the abstract, the same; its manifestation depends upon the function of the organ, and distinguishes muscle from nerve, nerve from gland-tissue.† Contraction is the manifestation of the function of muscle; sensation and the conduction of impressions, that of nerve; (these latter of course inappreciable to the observer, except in their results;) secretion, that of gland-tissue; etc.‡ Irritation, therefore, produces different and various phenomena, according to the organ or tissue upon which its influence is exerted.

* Joseph Hyrtl, Anatomie, etc.

† "If we speak of the life of the individual parts of a body, we must also know in what way life manifests itself, and whereby it is essentially characterized." [Virchow, Cellular Pathology, p. 23.

‡ It has been objected to the statement that muscle poseesses the peculiar property of contractility, that no new idea is advanced thereby, as the contractility is simply the manifestation of irritation. [Hodge, *opus citat.* p. 21.] Granting the fact that contractility is but the manifestation of due stimulus, it is difficult to see why it is therefore any the less a property, and, as found in muscle, both a peculiar and vital property. A property may be either active or passive; the former is called a *faculty*, the latter a *capacity*. Irritability, (excitability,) a property possessed by all tissues, is purely passive in its nature; the varied manifestations resulting therefrom are active and peculiar, and are individual "properties," in the strict sense of the word.

Further, molecular vitality has, of course, its grades, as well as its characteristic distinctions. It is much less, for example, in cartilage than in muscle. But it nevertheless exists in all, and in the fluids of the body as well as in the solids. As molecular, or cellular, vitality is the basis of all activity in the muscular or nerve-fibre, so does it animate the blood-corpuscle in the discharge of its individual functions. What these functions are, we may not be able, in the present state of our knowledge, fully to say, as their performance does not appeal directly to our physical senses. But no one doubts that the blood-corpuscle possesses the faculty of performing such functions, and, at the same time, a capacity for irritation. And it is because we observe that chief and most constantly existing pathological condition, inflammation, to be invariably characterized by disturbance in the complemental relation between these scavengers of waste and purveyors of repair on the one hand, and the ultimate cell constituents of the tissues on the other, that we can most properly give to this condition the comprehensive definition, "a lesion of nutrition."

The amount and kind of irritation, therefore, necessary to call into play the functions of different organs, varies as much as the resultant phenomena. The prick of a steel needle, which may be sufficient to cause a muscle to contract, will, perhaps, be without perceptible effect upon a nerve. But a more powerful stimulus, such as electricity, will produce the corresponding manifestation of function in the latter. Whatever may be the necessary stimulus, whether mechanical, chemical, or electrical, the physiological fact of molecular (cellular) organic life, proven by them, remains the same.

It is much to be regretted that so great confusion of ideas should have prevailed in regard to the nervous system. No part of physiology is so chary of affording definite ideas regarding cause and effect; nowhere in the animal organism is the *post hoc* so liable to be confounded with the *propter hoc*. The physiology of the nervous system, lying, as it does, upon the confines of the physiologist's domain, has always stood in danger of being obscured by the mist of metaphysics, in regard to all symptoms usually considered indicative of life. The fault here, we apprehend, lies not so much with physiology as with psychology; the cultivators of the latter science not sufficiently realizing the necessity of an acquaintance with the former, in order to proceed upon sound bases and from correct premises. The confusion is not to be wondered at, when one considers the limited nature of human knowledge, and the inadequacy of language to express that which is known. But it is not germane to our present purpose to enter at all upon so broad a field of inquiry and research. All that it is necessary to bear in mind here, is the fact that the nerves, in common with all organs, have a physiological or organic life *per se;* and although they are, through the cerebrum as a grand source, most intimately connected with the functions of that mysterious essence which we call mind, yet are they physiologically in no way dependent thereon.

The reflex action of the nerves is dependent on this power resident in the spinal cord, and mental consciousness or perception is not at all essential to its performance.* Further, it is important to remember that the contractility of muscle is not dependent upon the integrity of the nerves. Other causes, entirely distinct from the stimulus of innervation, can operate directly in arousing the dormant force of muscle. This is implied in what is said above of molecular (cellular) organic vitality. It is conclusively proven by experiments with the woorara poison, an agent which, although it completely destroys the vitality of the nerves, leaves untouched the muscular fibre. So strong is this proof, that even when the nerves refuse to respond in the slightest degree to the most powerful chemical and electrical stimuli, the muscles have been found awake to the slightest mechanical irritation.†

* The term "sensitive," applied to the afferent nerves, is open to some objection, on the ground of inaccuracy and ambiguity. It is manifestly incorrect to say, that an animal has lost *sensation*, and yet that the *sensitive* nerves still retain their integrity. Physiologically, then, there are sensitive (afferent) nerves in the ganglionic system of organic life. But this is not the general acceptation of the term "sensitive." At the same time, the proper distinction between sensation and consciousness is too well understood to need more than an allusion.

† Vid. Virchow, Cellular Pathology, p. 333.

We find, then, that all organized tissues in a state of nutrition have a molecular (cellular) life, manifested by the performance of functions peculiar to each, on the application of due and direct stimulus. It seems more than probable, that the seat of this molecular vitality is in the characteristic cell of the tissue, and, hence, the term cellular has been used above, as synonymous with, and explanatory of, the sense in which molecular is here to be understood.*

The basis of organization in all tissues is, necessarily, nutrition. This exists, of course, as well in the fœtus prior to birth as thereafter. Hence, the distinction made by Dr. DENMAN† between uterine and extra-uterine life seems to be, in this regard, a useless one. For all purposes upon which the properties belonging to organized tissues depend, life, whether intra or extra-uterine, is one and the same.

The case of Fish, or Fisher, *vs.* Palmer, in Beck, Medical Jurisprudence, vol. i., p. 410, first prompted these remarks. Here, the question turning upon the life of an infant at the time of birth, it was decided in the affirmative, on the simple evidence of servants that there were manifested "convulsive movements and twitchings of the lips" on the child being put into a warm bath. That the muscular contraction alluded to is strong evidence, if not proof, of *recent* life, I would by no means dispute. But it is consistent to suppose a case, in which a child, living a short time previous to birth, has died just before, or during, delivery. In such a case, is it not strictly in accordance with the teachings of physiology to find that the organized structures, muscle particularly, should retain sufficient organic vitality, resulting from the nutrition already received and the elements of which remain, to manifest the property of irritability on the application of a stimulus? Nay, more, should we not expect to find this?

* Since writing this article, I have seen in the *Am. Journal of Med. Sci.* for April, 1866, p. 511, a notice of Balbiani's researches. These investigations, however indefinite they may at present be, probably contain the germ of a truth that will ere long be universally recognized.

† *Fish* v. *Palmer*, Beck. Med. Jurisp., vol. i., p. 411.

In the case of Fish, or Fisher, *vs.* Palmer, it would, therefore, seem that the evidence of the two servants went negatively to show that the child was born dead; as it is stated, that beyond the convulsive twitching and tremulous motion of its lips, "it never manifested any other sign of life." Granting that the child was alive just previous to its entrance into the world, it is reasonable, and in accordance with our knowledge upon the subject, to conclude that the "convulsive twitching and tremulous motion of its lips" might very naturally have been caused by the bath of water into which the child was placed.

The object of these remarks is to show the insufficiency of muscular contraction as a demonstrating evidence of the presence of life—life, that is, in the common acceptation of the term. It is, therefore, in the case alluded to, the absence of any other sign of life whatever, which leads to the opinion that it was still-born.

It is, of course, impossible, in the present state of our knowledge, and it may always be so, to draw a definite line of demarcation, showing where life, as manifested by the presence of an immortal essence, (mind or soul,) begins. It is manifestly incorrect to consider "conscious existence" as a proper definition of this life, for who can prove that the infant, on taking its first breath, is conscious of anything?* This act may not only be independent of consciousness, (that is automatic,) but it may be a purely physiological one in itself, caused by the stimulus of the air or water upon the periphery of afferent nerves. That is, it may be eccentric; whereas automatic phenomena are (usually) centric in their causation. The maintainance and continuance of respiration is, of course, dependent upon the presence of that essential force which constitutes life; but who shall say what are the sure characteristics thereof; its (so to speak) physiognomonic evidences.

* It might at first seem that sleep affords sufficient argument against conscious existence as definitive of life. But it is probable that in strictness, consciousness exists even in the most profound sleep, although the brain, as a physical organ, may not be in a condition to manifest that consciousness to our perception at the time, or to our memory afterwards.

A case in point is related in the *American Journal of Medical Sciences*, for April, 1866. A woman, who was suckling a child ten months old, aborted with a fœtus of "from $6\frac{1}{2}$ to 7 inches in length, and, probably, of not more than five months' gestation." On removing the fœtus, the attending surgeon (Dr. J. D. MOORE) placed it partially enclosed on the membrane, and with the placenta still attached, in a vessel of water, the mother at that time requiring all his attention. "In about twenty minutes, I took it from the vessel, where it had been completely covered with fluid, in order to examine it, and, while taking its measurement, saw, distinctly, pulsation both in the thorax and fontanelles, and, after watching it for a few seconds, saw it open its mouth and make inspiratory movements, as if gasping for breath. The pulsation continued for some time, even after I had wrapped it in a napkin and carried it to my residence, (about five minutes' walk,) where it was also witnessed by several other medical men."

Can any one say what is the full import of these symptoms, and whether anything more than organic vitality can be predicated upon them? It is not purposed to follow out, here, this train of thought, which would necessarily lead to metaphysical abstractions of no practical value in the present stage of science. But, in a medico-legal point of view, it is important to have some definite and restricted definition of life, as distinguished from purely cellular vitality, which is simply a physiological condition. In cases like the one of Fish *vs.* Palmer, where there is necessarily room for much discussion, and in which, unless there be collateral evidence of a decisive nature, there must always remain doubt, it would be better to have some technical definition, which, however much it may be open to criticism, shall decide conclusively concerning it.

This is rendered all the more necessary, from the fact that there is no symptom at present known, which can be considered indubitable and conclusive proof of the presence of life. Numerous tests have been suggested and tried, but each one has been found open to more or less objection, the results being found, in certain cases, too fallacious to be reliable. The organs of digestion, the bladder, the liver, and the lungs have been made the bases of such tests.

We may pass all the former by, as entirely unreliable, except as substantiating other and stronger proofs, and come to the last named, *viz.*:—those by the lungs. Of these there are several—DANIELS' based upon the absolute gravity of the lungs, and the various changes induced by respiration; PLOUQUET'S, which takes into account the relative weight of the lungs to thal of the whole body, and the alteration in that weight by the act of breathing, etc.; and others.

The hydrostatic test (by floating) is the one which has met with the most favor, and which is still relied upon to a great extent. But this, like all the others, is by no means infallible, and numerous arguments might be adduced against its sufficiency. It will be enough here to remark, (1.) That the symptoms afforded by the hydrostatic test may be artificially produced; as, for example, by inflating, by decomposition, etc. (2.) On the other hand, some children have lived many hours, and yet but very few vesicles were found developed in the lungs.

All the tests which have been alluded to, have their foundation in the assumption that a new-born infant cannot breathe until after it has left the uterus. But the recorded occurrences of the *vagitus uterinus* are sufficient to show that this assumption is not borne out by fact. Hence, even if we possessed a satisfactory and reliable test for the occurrence of respiration, the fact that this had taken place, would not be enough to establish proof of extra-uterine life. It is, of course, extra-uterine life which must be proven, as there are many circumstances which may occur during labor sufficient to destroy life. The legal bearing of these points is obvious; as, for example, in cases of suspected infanticide.

The law of France makes (one?) full and complete respiration its legal definition of life. We have already seen some of the difficulties which must attend an attempt at proving this to have taken place, in cases where doubt exists. In addition to what has been mentioned, there is the fact that many children do not breathe for some time after birth, and yet live. With this fact every practitioner of obstetrics is, more or less, practically familiar.

The tendency of our common-law authorities, at present, is to require that an "independent circulation and existence" shall be proven, in order to constitute life.* The term "existence" implies nothing more than an abstract, passive condition; if independent, characterized by independent circulation and respiration, then the presence of active life may be considered as proven. From what has been said above, it will be seen that this definition is really the only admissible one, so far as our present knowledge goes.

Under this definition, and covered by it, come the relevant questions of "viability" and "adequate vitality." If a fœtus has not yet attained that age and growth (development) which is necessary for the continuance of its life out of the uterus, it has not the faculty of independent circulation and respiration. So, too, in cases where absence of the skull, or of the brain, (anencephali,) fissures of the spine, or similar want of development, would show absolute inability for the continuance of life. With these points we have nothing here to do, as they are subordinate to the question of the evidences of life as an independent existence.

Enough has been said to show the difficulty and uncertainty of this question. Its importance will not be gainsayed, although, fortunately, the cases in which the practitioner is called upon to decide such questions are comparatively infrequent. But none the less, therefore, is it incumbent on the medical man to study well the boundaries and distinctive features which may at any time have to decide important questions affecting humam happiness, property, and life.

The following points, then, with regard to this subject, may be considered as established:—

1. Every organ in the body is endowed with the property of irritability.

2. This property is resident in the organic molecular cell of

* Wharton, Criminal Law, §874, where a full list of authorities are referred to, and may be found specified. Cf. §§710 and 942.

the tissue. These cells may, therefore, be considered as so many vital units of organization.

3. The phenomena manifested by each organ, on the occurrence of an irritation, constitute its individual and distinguishing properties.

4. These properties of the tissues are, in themselves, considered independent (*a.*) of consciousness; (*b.*) of the *vis incita*, termed "life;" and (*c.*) of nervous integrity, except, of course, with regard to the nerve-tissue itself, as a distinct organ.

5. The sole requisite seems to be the presence of active organization—elements of nutrition not yet yielded up to chemical laws.

6. The occurrence of muscular contraction, or performance of other like functions, must, therefore, be considered as simply dependent upon the organic vitality resident in the cells of the tissue or organ; and the performance of such functions cannot be held as evidence of anything more than cellular vitality.

7. Hence, in the present state of our knowledge, nothing less than an independent circulation and respiration can be accepted as constituting "life," in the ordinary acceptation of the term. If these can be proven to have existed, even for an indefinitely short time, the question of life may be considered as decided in the affirmative.

Much more space than is here permitted, would be required to do justice to the above subject. In what has been said, the aim has not been to present things original or unknown to the profession, but to suggest further thought and inquiry upon a subject which is, perhaps, too apt to be neglected in the round of daily practice.

